# Psychiatric inpatient expenditures and public health insurance programmes: analysis of a national database covering the entire South Korean population

**DOI:** 10.1186/1472-6963-10-263

**Published:** 2010-09-07

**Authors:** Woojin Chung

**Affiliations:** 1 Department of Health Policy and Management, Graduate School of Public Health and Institute of Health Services Research, Yonsei University, Seoul 120-752, Republic of Korea

## Abstract

**Background:**

Medical spending on psychiatric hospitalization has been reported to impose a tremendous socio-economic burden on many developed countries with public health insurance programmes. However, there has been no in-depth study of the factors affecting psychiatric inpatient medical expenditures and differentiated these factors across different types of public health insurance programmes. In view of this, this study attempted to explore factors affecting medical expenditures for psychiatric inpatients between two public health insurance programmes covering the entire South Korean population: National Health Insurance (NHI) and National Medical Care Aid (AID).

**Methods:**

This retrospective, cross-sectional study used a nationwide, population-based reimbursement claims dataset consisting of 1,131,346 claims of all 160,465 citizens institutionalized due to psychiatric diagnosis between January 2005 and June 2006 in South Korea. To adjust for possible correlation of patients characteristics within the same medical institution and a non-linearity structure, a Box-Cox transformed, multilevel regression analysis was performed.

**Results:**

Compared with inpatients 19 years old or younger, the medical expenditures of inpatients between 50 and 64 years old were 10% higher among NHI beneficiaries but 40% higher among AID beneficiaries. Males showed higher medical expenditures than did females. Expenditures on inpatients with schizophrenia as compared to expenditures on those with neurotic disorders were 120% higher among NHI beneficiaries but 83% higher among AID beneficiaries. Expenditures on inpatients of psychiatric hospitals were greater on average than expenditures on inpatients of general hospitals. Among AID beneficiaries, institutions owned by private groups treated inpatients with 32% higher costs than did government institutions. Among NHI beneficiaries, inpatients medical expenditures were positively associated with the proportion of patients diagnosed into dementia or schizophrenia categories. However, for AID beneficiaries, inpatient medical expenditures were positively associated with the proportion of all patients with a psychiatric diagnosis that were AID beneficiaries in a medical institution.

**Conclusions:**

This study provides evidence that patient and institutional factors are associated with psychiatric inpatient medical expenditures, and that they may have different effects for beneficiaries of different public health insurance programmes. Policy efforts to reduce psychiatric inpatient medical expenditures should be made differently across the different types of public health insurance programmes.

## Background

Medical spending on psychiatric hospitalization has been reported to impose a tremendous socio-economic burden on many countries [1-5] and South Korea (hereafter, Korea) is not an exception. In Korea, the average length of stay for psychiatric inpatients was 89.8 days in 2005, which was more than 12 times as long as that of inpatients in the United States [6,7], and the rate of increase in psychiatric inpatient spending was, on average, about 13% per year during the period from 2000 to 2006. Moreover, surprisingly, psychiatric inpatient care differs significantly across different types of public health insurance programmes. Despite the fact that the number of beneficiaries of a public aid system (the National Medical Care Aid, AID) is about 4% of those of a public insurance system (the National Health Insurance system, NHI), Korea's total expenditures on psychiatric inpatient care of AID beneficiaries were more than 114% of those of NHI beneficiaries in 2006 [8,9]. However, policy makers in Korea are yet to identify a suitable measure to control medical expenditures (hereafter MEs) for psychiatric inpatients [10]. The major reason for this might be the lack of study on the factors affecting psychiatric MEs in Korea. In particular, from the policy perspective, separate analysis of each type of public health insurance programme and accompanying comparisons of results of these programmes seems to be desperately needed. It may be possible to draw some policy implications from previous studies conducted in other countries; however, this seems very difficult to achieve. Although numerous studies have dealt with similar topics throughout the world, they have the following limitations: only a few regions in a country were analysed [11-21]; only a few categories of age were considered; limited psychiatric diseases were analysed [16,17,22-24]; only one type of public health insurance programme was sampled [18,25-28]; and only descriptive analyses were conducted [18-20,24-31].

Therefore, the purpose of this study is to explore factors affecting psychiatric inpatient MEs and test whether the extent to which these factors affect psychiatric inpatient MEs varies across different types of public health insurance programmes. The reasons why I focused on the medical expenditure, rather than demand, are as follows: first, from the health policy perspective, a study of medical expenditures and factors affecting them is of great importance in the design of policies to contain these expenditures. Second, it is very difficult to correctly identify the factors affecting demand using the market outcome data, because of the identification problem [32]. Third, factors affecting medical expenditures have attracted much attention, even academically [33-35].

To achieve my purpose, I employed Korea's national databases covering all inpatients nationwide and used multilevel analyses in order to adjust for potential clustering within a medical institution. In light of this study's purposes, Korea is a country that provides favorable system characteristics, because it implements two types of public health insurance programmes: NHI for non-poor persons and AID for the poor, covering all of Korea's population, which exceeds 48 million people. To explain these national health security programmes briefly, both programmes provide for the same types of medical services and all the different medical institutions are required to admit both types of beneficiaries. AID beneficiaries receive financial assistance to cover more than 85% of their expenditures from the government, whereas NHI beneficiaries receive financial assistance to cover, on average, 73% of their expenditures from the National Health Insurance Corporation. The amount that patients are required to pay as an out-of-pocket expense increases from primary care institutions to tertiary care institutions. Even for the same type of care, the fees that medical institutions receive as reimbursement for their costs increase from primary care institutions to tertiary care institutions [9,36]. Meanwhile, reimbursement varies for the same treatment depending on the institutions at which inpatients are treated and on the types of public health insurance programmes in which inpatients are enrolled.

## Methods

### Data source and study sample

From national reimbursement claim databases, I selected all claims for inpatient care between January 1, 2005 and June 30, 2006, which included psychological and behavioural disorders as the principal diagnosis code (F code) of the ICD-10 codes (1,131,346 claims). Information about the medical institutions providing the inpatient care was obtained from national databases of medical resources and was merged into the chosen claims. After sorting the datasets, I obtained information on 160,465 inpatients who accounted for 93.08% of all claims. F code is classified into nine sub-diagnosis codes, F0-F9, and each psychiatric inpatient was determined to be, on average, associated with 1.09 sub-diagnosis codes. Therefore, we defined the principal sub-diagnosis of each inpatient by identifying the sub-diagnosis code that maximally contributed to the total length of stay of each inpatient. To examine the relationships between institutional characteristics and inpatient MEs, I identified the medical institution at which each inpatient stayed for the longest period of time for treatment of his or her principal sub-diagnosis and incorporated the characteristics of that institution into further analyses. The subjects included in this study were treated by a total of 471 institutions. This study was approved by the Institutional Review Board of Yonsei University Health System.

### Measures and variables

The dependent variable is total medical expenditure per inpatient admitted because of a psychiatric condition between 1^st ^January 2005 and 30^th ^June 2006. The MEs were expressed in 1000 units of Korean Won (KRW). Explanatory variables included both inpatient and institution characteristics. Each inpatient was either an NHI beneficiary or an AID beneficiary. Patient ages on January 1, 2005, were categorized into six ranges: <20 years; 20-29 years; 30-39 years; 40-49 years; 50-64 years; and ≥ 65 years. The principal sub-diagnosis was expressed according to ICD-10 codes: F0, organic mental disorders (including symptomatic disorders and dementia); F1, mental and behavioural disorders due to the use of psychoactive substances; F2, schizophrenia, schizotypal and delusional disorders; F3, mood [affective] disorders; F4, neurotic, stress-related, and somatoform disorders; F5, behavioural syndromes associated with physiological disturbances and physical factors; F6, disorders of adult personality and behaviour; F7, mental retardation; F8-9, others.

Characteristics of the medical institutions were also included. Medical institutions at which an inpatient stayed for treatment of a principal sub-diagnosis were grouped into seven categories: psychiatric clinic, psychiatric hospital, (non-psychiatric) clinic, hospital, general hospital, tertiary care hospital, and long-term care hospital. In Korea, according to medical care laws, all types of medical institutions are allowed to admit inpatients as long as they are equipped with beds [37]. Clinics, hospitals, and general hospitals are mainly classified by the number of beds: fewer than 30 beds for clinics, 30 to 99 beds for hospitals, and more than or equal to 100 beds for general hospitals. However, in addition to the required number of beds, general hospitals must be equipped with a set of specialties and specialists; for example, a general hospital having 100-299 beds must have at least three required specialties such as internal medicine, general surgery, paediatrics, and obstetrics, and their related specialists. Tertiary care hospitals are mainly associated with universities and educate medical professionals, conduct research, and provide medical care. Psychiatric medical institutions must treat a fixed proportion of patients with psychiatric illness relative to the total numbers of patients. The owners of institutions were categorized into government, private groups, and private persons. The places at which medical institutions were located were designated as either a metropolis, a small/medium city, or a rural area. In order to categorise institutions in regards to their number of beds, I took the following four steps. First, I identified the medical institution at which each inpatient stayed for the longest period of time for treatment of his or her principal sub-diagnosis. Second, because each institution identified for each inpatient has a different number of beds, the number of beds at each institution identified for each inpatient was defined as the "number of beds" variable. Third, I sorted institutions based on the "number of beds" variable in ascending order. Finally, I divided the "number of beds" variable into four categories so that each category (< 260, 260-399, 400-626 and ≥ 627 beds) included one quarter of the total number of psychiatric inpatients (including both NHI and AID beneficiaries).

Regarding the human resources of a medical institution, I considered four kinds of full-time healthcare and social service professionals at a medical institution: physicians, nurses, nursing assistants, and others. The number of each kind of professional was divided by 100 beds for each medical institution. To analyse the effect of inpatient composition at each institution on inpatient ME, I added four variables namely, the proportion of inpatients with a psychiatric diagnosis that were; male, ≥ 65-year-old, diagnosed into categories F0 or F2, and, were receiving AID. The reason why I am interested in the proportion of F0 and F2 diagnoses is that these diagnoses, which include dementia and schizophrenia, were reported to be the largest contributor to psychiatric health care costs in Korea[38].

### Analytic procedures

My analysis is five-fold. First, I conducted a principal component analysis to reduce colinearity among four separate variables comprising human resources and extracted components with eigenvalues greater than 1.0. The first and only principal component that increased with values of all four original variables had a uniquely high eigenvalue (2.95) and accounted for 74% of the variation in all four of the original variables. In subsequent statistical analyses, the original four variables were replaced by this principal component, Score 1 [39,40]. Second, I tested the differences in characteristics of both patients and medical institutions between NHI and AID beneficiaries by using a t-test. Third, I investigated patient and institutional characteristics associated with patient MEs. Based on skewness, kurtosis, and the Kolmogorov-Smirnov *D *statistic (details available on request) [41], patient MEs were not normally distributed. Therefore, I used a series of non-parametric tests: the Mann-Whitney test, the Kruskal-Wallis test, the Pearson correlation test, and the Friedman's two-way non-parametric analysis of variance (ANOVA) test. Fourth, I employed the Box-Cox family of power transformations in order to obtain an approximate normal distribution of residuals in the multivariate linear regression model [42]. For obtaining the power parameter (λ) for each beneficiary group, I used the maximum likelihood estimation method along with a grid search.

The last step involved the multilevel structure of observations in which the number of inpatients treated at the same institution was relatively large and varied significantly across institutions (mean, 340.69; range, 1-2, 607). This implied that patients were likely to be correlated among one another within the same institution. In the presence of this correlation, it can be difficult for standard linear regression analyses to satisfy classical regression assumptions. In particular, this correlation will likely lead to a violation of the assumption of uncorrelated errors [43]. Therefore, I used multilevel analyses (linear mixed models with random intercepts) with two levels: the patient-level and the institution-level. To summarize, the results of my final regression analyses were obtained from the following three steps. I first transformed the ME variable of each psychiatric inpatient using Box-Cox transformation procedures. Then, I regressed the each inpatient Box-Cox transformed ME variable on inpatient and institutional characteristics through multilevel analyses. Finally, to determine whether the effects of inpatient and institutional characteristics on inpatient medical expenditures differ for beneficiaries of different public health insurance programmes, I converted each estimated coefficient to the rate of change (%) evaluated at the median value of inpatient MEs. This analysis was conducted separately for each public health insurance programme. Values of *p *< 0.05 were considered statistically significant. The package SAS version 9.1 was used for statistical analysis.

## Results

### Differences in patients and institutional characteristics: univariate analyses

Most characteristics for psychiatric inpatients were significantly different between NHI beneficiaries and AID beneficiaries (Table 1). Particularly, AID beneficiaries were more likely to be male, aged 40-49 years old, and diagnosed into schizophrenia, schizotypal, and delusional disorders (F2) diagnosis than NHI beneficiaries.

**Table 1:** Inpatient characteristics: Public insurance (NHI) beneficiaries versus public aid (AID) beneficiaries (N = 160,465).

	NHI beneficiary		AID beneficiary		
	(n = 92,959)		(n = 67,506)		
		
Characteristics	n	%	n	%	p-value
Gender					
Female	42,719	46.0	22,630	33.5	<.0001
Male	50,240	54.0	44,876	66.5	<.0001
Age (years)					
<20	4,274	4.6	1,088	1.6	<.0001
20-29	11,860	12.8	3,689	5.5	<.0001
30-39	18,920	20.3	13,968	20.7	.0973
40-49	20,472	22.0	23,550	34.9	<.0001
50-64	20,361	21.9	15,601	23.1	<.0001
≥ 65	17,072	18.4	9,610	14.2	<.0001
Principal sub-diagnosis					
F0	9,175	9.9	5,468	8.1	<.0001
F1	23,544	25.3	19,266	28.5	<.0001
F2	26,550	28.6	33,483	49.6	<.0001
F3	23,249	25.0	4,736	7.0	<.0001
F4	7,602	8.2	742	1.1	<.0001
F5	470	0.5	50	0.1	<.0001
F6	900	1.0	572	0.9	.0122
F7	685	0.7	2,914	4.3	<.0001
F8-9	784	0.8	275	0.4	<.0001

Note: Medical expenditures are expressed in 1000 units of Korean Won (KRW); USD 1 = KRW 948.80 (June, 2006); the p-value is based on the t-test.

Institutions that had treated NHI-enrolled inpatients were significantly different from those that had treated AID-enrolled inpatients (Table 2). Particularly, AID beneficiaries were more likely than NHI beneficiaries to be treated at a psychiatric hospital, at an institution in a non-metropolitan area, or at an institution with 260-626 beds.

**Table 2:** Inpatient characteristics across institutional characteristics: Public insurance (NHI) beneficiaries versus public aid (AID) beneficiaries (N = 160,465).

	NHI beneficiary		AID beneficiary		
	(n = 92,959)		(n = 67,506)		
		
Characteristics	N	%	N	%	p-value
Type					
Psychiatric clinic	7,659	8.2	2,999	4.5	<.0001
Psychiatric hospital	8,348	9.0	14,458	21.4	<.0001
Clinic	2,102	2.3	1,317	2.0	<.0001
Hospital	34,968	37.6	40,103	59.4	<.0001
General hospital	18,971	20.4	7,386	10.9	<.0001
Tertiary care hospital	18,853	20.3	876	1.3	<.0001
Long-term care hospital	2,058	2.2	367	0.5	<.0001
Ownership					
Government	9,288	10.0	7,900	11.7	<.0001
Private group	57,498	61.8	41,037	60.8	<.0001
Private person	26,173	28.2	18,569	27.5	.0043
Location					
Metropolis	44,542	47.9	21,432	31.7	<.0001
Small/medium city	41,174	44.3	34,405	51.0	<.0001
Rural area	7,243	7.8	11,669	17.3	<.0001
Number of beds					
<260	24,442	26.3	15,110	22.4	<.0001
260-399	17,580	18.9	22,732	33.7	<.0001
400-626	22,462	24.2	17,574	26.0	<.0001
≥ 627	28,475	30.6	12,090	17.9	<.0001

Note: p-value is based on the t-test.

### Factors influencing inpatient ME: univariate and bivariate analyses

The median value of inpatient MEs among AID beneficiaries was three times as high as that among NHI beneficiaries (*p *< 0.001) (Table 3). Inpatient MEs differed according to gender, age, or sub-diagnosis (*p *< 0.001). Male patients tended to incur higher MEs than their female counterparts. Among all sub-diagnoses, F2 involved the highest MEs, whereas F4 produced the lowest MEs. The Friedman tests showed that in determining MEs, the type of public health insurance programme interacted significantly with every inpatient characteristic (*p *< 0.001).

**Table 3:** Inpatient characteristics associated with inpatient medical expenditures, univariate and bivariate analyses: public insurance (NHI) beneficiaries versus public aid (AID) beneficiaries (N = 160,465).

	NHI beneficiary		AID beneficiary		
	(n = 92,959)		(n = 67,506)		
		
Characteristics	Median	(Range)	Median	(Range)	p-value
Medical expenditure	1,743.9	(0.4-48222.6)	5324.6	(8.6-26789.5)	<.0001*
					
Gender	<.0001*		<.0001*		
Female	1,555.8	(0.4-39236.9)	4,943.9	(8.6-26789.5)	<.0001***
Male	1,938.8	(1.4-48222.6)	5,441.6	(8.6-25099.7)	
					
Age (years)	<.0001**		<.0001**		
<20	1,433.4	(4.5-48222.6)	1,667.2	(8.6-16738.4)	
20-29	2,008.8	(5.6-31954.3)	4,683.0	(17.1-21430.2)	
30-39	2,006.2	(0.4-34840.7)	5,359.2	(8.6-23561.7)	<.0001***
40-49	1,626.7	(3.5-40935.2)	5,650.8	(8.6-25099.7)	
50-64	1,624.2	(0.8-39622.3)	6,344.2	(17.1-26789.5)	
≥ 65	1,678.1	(1.4-36711.0)	3,911.6	(8.6-19506.7)	
					
Principal sub-diagnosis	<.0001**		<.0001**		
F0	2,073.2	(4.0-36711.0)	3,696.0	(17.1-19506.7)	
F1	1,717.6	(1.4-35177.6)	4,165.1	(8.6-21430.2)	
F2	2,762.4	(4.2-40935.2)	8,561.1	(8.6-25099.7)	
F3	1,390.9	(0.4-27993.7)	1,941.3	(8.6-26789.5)	<.0001***
F4	619.7	(3.5-30507.9)	755.6	(17.1-16062.3)	
F5	912.9	(6.7-17676.2)	1,177.9	(30.8-14762.8)	
F6	1,653.3	(24.7-25376.7)	3,487.4	(17.1-17712.4)	
F7	1,986.2	(18.7-48222.6)	6,016.2	(30.8-21383.7)	
F8-9	1,164.5	(4.5-24773.8)	1,570.8	(17.1-16209.0)	

Note. Medical expenditures are expressed in 1000 units of Korean Won (KRW); USD 1 = KRW 948.80 (June, 2006); *p-values are based on the Mann-Whitney test; **p-values are based on the Kruskal-Wallis test; ***p-values are based on the Friedman's two-way non-parametric ANOVA test for the interaction terms between the public health insurance programme and each characteristic.

Inpatient MEs also differed across institutional characteristics such as type, ownership, location, and the number of beds (*p *< 0.001) (Table 4). For example, compared with inpatients staying at tertiary care hospitals, the highest median values of MEs were found in those staying at long-term care hospitals among NHI beneficiaries, but those staying at psychiatric hospitals among AID beneficiaries the values were two times and seven times higher than those at tertiary care hospitals. Additionally, institutional characteristics regarding human resources and patient composition were significantly associated with MEs but had slight differences between NHI-enrolled inpatients and AID-enrolled inpatients (*p *< 0.001). For example, Score 1 (representing human resources) showed negative relationships with MEs for both NHI and AID beneficiaries, whereas in the proportions of male patients, those diagnosed with F0 or F2 codes and those receiving AID showed positive relationships with MEs. The Friedman tests showed that in determining patient MEs, the type of public health insurance programme interacted significantly with institutional characteristics such as type, ownership, location, and the number of beds (*p *< 0.001).

**Table 4:** Institutional characteristics associated with inpatient medical expenditures, univariate and bivariate analyses: Public insurance (NHI) beneficiaries versus public aid (AID) beneficiaries (N = 160,465).

	NHI beneficiary		AID beneficiary		
	(n = 92,959)		(n = 67,506)		
		
Characteristics	Median	(Range)	Median	(Range)	p-value
Type	<.0001**		<.0001**		
Psychiatric clinic	1,437.0	(4.3-27262.6)	2,762.2	(30.8-19153.0)	
Psychiatric hospital	2,586.6	(27.3-30723.7)	11,348.8	(29.7-25099.7)	
Clinic	2,041.7	(5.6-22704.3)	3,110.8	(30.8-16953.5)	<.0001***
Hospital	2,100.7	(1.4-48222.6)	4,892.3	(8.6-23561.7)	
General hospital	1,256.0	(0.4-40935.2)	3,770.7	(26.0-26789.5)	
Tertiary care hospital	1,583.8	(7.7-39622.3)	1,709.1	(30.8-16740.0)	
Long-term care hospital	2,837.5	(18.5-36711.0)	2,632.1	(29.8-19506.7)	
					
Ownership	<.0001**		<.0001**		
Government	2,119.8	(4.0-48222.6)	2,570.0	(8.6-20333.3)	
Private group	1,701.0	(0.4-40935.2)	7,252.0	(26.0-26789.5)	<.0001***
Private person	1,715.6	(1.4-31442.3)	3,942.4	(10.5-21744.0)	
					
Location	<.0001**		<.0001**		
Metropolis	1,656.4	(3.5-48222.6)	4,281.2	(8.6-21383.7)	
Small/medium city	1,755.2	(0.4-36711.0)	5,965.5	(8.6-26789.5)	
Rural area	2,478.3	(1.4-29575.9)	5,594.3	(17.1-23561.7)	
					
Number of beds	<.0001**		<.0001**		
<260	1,710.5	(4.1-48222.6)	3,648.6	(17.1-19775.5)	
260-399	1,906.5	(0.4-33640.2)	5,353.5	(8.6-21744.0)	<.0001***
400-626	1,717.6	(4.1-40935.2)	6,208.4	(8.6-23561.7)	
≥ 627	1,718.0	(0.8-39622.3)	6,995.8	(8.6-26789.5)	

	R	p-value	R	p-value	p-value**

Human resources: Score 1	-0.182	0.000	-0.231	0.000	<.0001
Pro of male patients	0.154	0.000	0.078	0.000	<.0001
Pro of patients ≥ 65-year-old	0.052	0.000	-0.080	0.000	<.0001
Pro of patients diagnosed into F0 or F2	0.187	0.000	0.161	0.000	<.0001
Pro of patients receiving AID	0.173	0.000	0.272	0.000	<.0001

Note: Medical expenditures are expressed in 1000 units of Korean Won (KRW); USD 1 = KRW 948.80 (June, 2006); pro denotes proportion; **p-values are based on the Kruskal-Wallis test; ***p-values are based on Friedman's two-way non-parametric ANOVA test for the interaction terms between the public health insurance programme and each characteristic. R denotes the Pearson correlation coefficient; Score 1 = 0.54 × Number of physicians per 100 beds + 0.55 × Number of nurses per 100 beds + 0.33 × Number of nursing assistants per 100 beds + 0.55 × Number of other healthcare and social service professionals per 100 beds.

### Adjusted factors influencing inpatient MEs: multi-level analyses

According to the results from all the univariate and bivariate tests conducted above, I confirmed that a study of factors influencing psychiatric inpatient MEs should be conducted separately for NHI beneficiaries than those for AID beneficiaries. Therefore, I obtained the Box-Cox power parameters to transform inpatient ME (λ = 0 among NHI beneficiaries and λ = 0.5 among AID beneficiaries) for each beneficiary group. Then, after conducting both standard linear regression analyses and multilevel analyses, I compared the results of both types of analyses and summarized them as follows. First, the deviance of multilevel analyses is significantly lower than that of standard linear regression analyses for each beneficiary group (*p *< 0.001). Similarly, the multilevel analyses had lower values for both the Akaike Information Criterion and the Schwarz's Bayesian Information Criterion for each beneficiary group. Lastly, the interclass correlation, which tells us what portion of the total variance occurs between medical institutions, was estimated to be 0.21 among NHI-enrolled inpatients and 0.27 among AID-enrolled inpatients, which is suggestive of considerable clustering of inpatient MEs within a medical institution [43]. These results indicate that the multilevel analyses fit significantly better than the standard linear regression analyses.

Table 5 shows the results of multilevel analyses, where, for expository convenience, the effect of each estimated coefficient on inpatient MEs was converted to the rate of change (%) evaluated at the median of MEs. The rate of change may explain how much inpatient MEs would change if a characteristic of interest changes from the concerned referent category to another category, all other things being equal. According to my results, male inpatients incur 12% higher MEs than their female counterparts among NHI beneficiaries and 10% higher MEs among AID beneficiaries. MEs of inpatients between the ages of 50-64 would be 10% higher among NHI beneficiaries and 40% higher among AID beneficiaries than those younger than 20 years. Inpatients with an F2 diagnosis were associated with the highest MEs among both beneficiary groups; compared with those diagnosed with F4 disorders, their MEs would be 83% higher among AID beneficiaries and 120% higher among NHI beneficiaries.

**Table 5:** Characteristics associated with inpatient (Box-Cox transformed) medical expenditures, multilevel analyses: Public insurance (NHI) beneficiaries versus public aid (AID) beneficiaries (N = 160,465).

	NHI beneficiary		AID beneficiary	
	(n = 92,959)		(n = 67,506)	
	
Characteristics	Rate of change (%)	p-value	Rate of change (%)	p-value
Gender				
Female (referent)				
Male	12.29	0.000	9.76	0.000
Age (years)				
<20 (referent)				
20-29	8.59	0.000	24.05	0.000
30-39	6.92	0.001	30.19	0.000
40-49	1.69	0.416	34.99	0.000
50-64	10.03	0.000	39.72	0.000
≥ 65	9.23	0.000	25.65	0.000
Principal sub-diagnosis				
F4 (referent)				
F0	75.69	0.000	44.84	0.000
F1	55.25	0.000	32.37	0.000
F2	120.10	0.000	82.77	0.000
F3	61.58	0.000	23.16	0.000
F5	21.39	0.000	-0.16	0.990
F6	59.80	0.000	31.10	0.000
F7	75.12	0.000	66.51	0.000
F8-9	37.30	0.000	45.55	0.000
Type				
General hospital (referent)				
Clinic	60.00	0.000	-3.73	0.774
Hospital	52.00	0.000	6.78	0.471
Tertiary care hospital	27.00	0.006	-7.63	0.557
Long-term care hospital	60.00	0.000	19.54	0.310
Psychiatric clinic	46.00	0.000	9.36	0.431
Psychiatric hospital	74.00	0.000	42.10	0.000
Ownership				
Government (referent)				
Private group	-1.80	0.825	32.19	0.000
Private person	-10.70	0.236	17.75	0.039
Location				
Metropolis (referent)				
Small/medium city	-1.78	0.686	3.92	0.369
Rural area	7.52	0.324	-2.30	0.741
Number of beds				
<260 (referent)				
260-399	4.09	0.540	11.44	0.067
400-626	10.47	0.164	11.02	0.128
≥ 627	4.83	0.534	1.96	0.824
Human resources: Score 1	0.03	0.216	-0.09	0.006
Pro of male patients	0.64	0.000	0.43	0.016
Pro of patients ≥ 65-year-old	0.24	0.106	0.09	0.593
Pro of patients diagnosed into F0 or F2	0.60	0.000	0.26	0.068
Pro of patients receiving AID	-0.05	0.649	0.42	0.002

Note: The estimated coefficients were converted to the corresponding rate of change evaluated at the median of medical expenditures among inpatients enrolled in each public health insurance programme; Score 1 = 0.54 × Number of physicians per 100 beds + 0.55 × Number of nurses per 100 beds + 0.33 × Number of nursing assistants per 100 beds + 0.55 × Number of other healthcare and social service professionals per 100 beds; pro denotes proportion.

Among NHI beneficiaries, compared with inpatients staying at general hospitals, those staying at the other medical institutions tended to incur higher MEs. In particular, psychiatric hospitals were found to be associated with the highest MEs among all types of medical institutions; for example, MEs of inpatients staying at psychiatric hospitals were 74% higher than those of inpatients staying at general hospitals. Meanwhile, among AID beneficiaries, the type of institutions showed no significant difference in ME across medical institutions except for psychiatric hospitals. Inpatients staying at psychiatric hospitals were found to be associated with the highest MEs; their MEs were 42% higher than those admitted to general hospitals. Additionally, ownership of medical institutions was a significant factor only among AID beneficiaries; MEs were 32% and 18% higher in medical institutions owned by private groups and by private persons, respectively, than in government-owned institutions.

Human resources of medical institutions tended to show a negative relationship with patient MEs among AID beneficiaries; an increase in Score 1 by 10% would decrease the MEs by 0.9%. Patient composition at a medical institution was also likely to affect patient ME. A 10% increase in the proportion of inpatients with a psychiatric diagnosis that were male was found to be associated with an increase in patient MEs of 6.4% among NHI beneficiaries and 4.3% among AID beneficiaries. A 10% increase in the proportion of patients diagnosed into the F0 or F2 categories was found to be associated with a 6% increase patient MEs among NHI beneficiaries, whereas a 10% increase in the proportion of psychiatric inpatients enrolled in AID was found to be associated with a 4.2% increase in patient MEs among AID beneficiaries.

## Discussion

### Public health insurance programme

The type of health care system has been reported to influence the provision of psychiatric services [21,44]. In Korea, high psychiatric inpatient MEs among AID beneficiaries are partially due to the inpatients' prolonged stay because of the defects in the health care system. For AID beneficiaries, both providers (medical institutions) and consumers (patients and their families) tend to delay patients' discharges. From the standpoint of providers, inadequate oversight of quality of care, lack of coordination of mental health care and a low per-diem rate may result in prolonging admissions [7,10,45]. Per-diem rates are typically low, but cover the average production costs (fixed costs plus variable costs) of most Korean medical institutions. Therefore, medical institutions, which must bear the burden of fixed costs associated with unoccupied beds, tend to admit psychiatric patients and keep them for long periods of time in order to minimize their fixed cost burdens, especially because AID beneficiaries are cared for under no or very low cost sharing structures [46]. Consumers are also often reluctant to be discharged. In Korea, psychiatric day care and non-medical services (like residential service and vocational rehabilitation) for AID beneficiaries are not subsidized by the government, and community-based mental health services are not well provided. When patients stay at home they and their families bear the full amount of the monetary and non-monetary burden of their care, whereas when they stay at medical institutions it is free or at a very low cost. These circumstances tend to facilitate institutionalization syndrome as experienced in many countries [10,45,46].

### Gender

Previous studies have shown that, on average, men receive less psychiatric treatment than women [47,48]. This observation may be related to the different types of mental disorders that men and women typically experience. For example, women tend to have a higher frequency of mood and/or anxiety disorders that are more likely to respond to psychiatric treatment than men [49,50]. In contrast to the previous findings, I found that males showed a greater medical use compared with female psychiatric inpatients in terms of inpatient medical expenditures among both NHI and AID beneficiaries. A further investigation confirmed that even in terms of the length of stay, male inpatients tended to stay 16% longer than female inpatients (details available on request). According to these findings, factors that have gone unnoticed might affect gender differences in psychiatric care utilization. One potential reason for this is related to the greater difficulties in caring for mentally ill males at home and at community-based facilities than there seem to be for their female counterparts in Korea. Having a mentally ill family member affects family functioning, which can lead to increased burden on other family members [51]. Men with psychiatric disorders are less likely to be tractable than women, so male patients are more likely to be excluded from care at home and from community-based facilities than are females. Another possible explanation for this gender difference involves homeless persons with psychiatric disorders. Due to the lack of community-based mental health facilities in Korea, central and local governments have institutionalized homeless patients with mental illnesses. The fact that the majority of homeless and mentally ill patients are men rather than women might partially explain why patient MEs were higher among men than among women [45]. Lastly, the differences in the types and composition of psychiatric sub-diagnoses might lead to gender differences in MEs. A study in the United States reported that men had higher rates of hospitalization than women for alcohol and drug disorders, whereas women had higher hospitalization rates for affective disorders [31]. However, the further analysis of the Kruskal-Wallis test revealed that in Korea, males are significantly associated with higher MEs than females both for inpatients with F1 sub-diagnosis related to alcohol and drug disorders and for inpatients with F3 sub-diagnosis related to affective disorders (*p *< 0.0001) (details available on request).

### Age

I found that psychiatric inpatient MEs were significantly associated with inpatient age among both NHI and AID beneficiaries. This is similar to findings seen in other countries [20,24,52]. In my study, particularly, inpatients aged 50-64 showed the highest MEs among inpatients for all categories of age. Despite the fact that there were no large differences in median MEs of inpatients younger than 20 between NHI and AID beneficiaries (KRW 1,433,400 vs. 1,667,200) (Table 3), compared with inpatients younger than 20, the adjusted MEs of inpatients aged 50-64 was 10% higher among NHI beneficiaries but 40% higher among AID beneficiaries (Table 5). Additionally, the pattern in the differences in MEs across age categories was quite different between NHI beneficiaries and AID beneficiaries. This indicates that the management of costs for psychiatric inpatients depending on a category of age should be implemented differently across public health insurance programmes.

### Sub-diagnosis

My findings that psychiatric sub-diagnoses are significantly associated with inpatient MEs are consistent with those from previous studies [19,28,31]. In particular, the effect of psychiatric sub-diagnoses on inpatient MEs showed the largest variance among all patient and institutional characteristics. MEs were highest for patients who were treated for schizophrenic disorder (F2) and mental retardation (F7) among both NHI and AID beneficiaries. Indeed, schizophrenia was reported to be one of the most expensive psychiatric disorders across the adult lifespan [20,24,52]. For non-elderly inpatients, medical spending was highest among those who were treated for schizophrenic disorder, other organic disorders or dementia [19]; and for elderly patients, medical spending was highest among patients who were treated for schizophrenic disorder, major depressive disorder, or bipolar disorder in Maryland state general hospitals in 1998 [19], and among Medicare beneficiaries in the United States [28].

### Institution type

My results indicate that inpatients of psychiatric hospitals tend to incur the highest costs. This is consistent with findings from a previous descriptive analysis [28]. Because psychiatric patients with a higher rate of comorbidity are often admitted to tertiary care hospitals, higher MEs among inpatients staying at psychiatric hospitals have attracted much attention. A potential reason for this might be related to the fact that as requested by the government, psychiatric hospitals often treat homeless, mentally ill patients, and those inpatients are unlikely to be discharged even after they are cured because of the lack of community-based mental facilities and also because of stigmas [45,53,54]. Another interpretation for higher MEs at psychiatric hospitals is because most psychiatric hospitals were built a long time ago, so there is an accumulated number of chronically ill inpatients suffering from regressive, psychiatric disorders [46,55]. Lastly, the higher MEs seen at psychiatric hospitals might be due to the reimbursement fee schedule instituted by public health insurance systems. In Korea, all medical institutions are forced to obey public health insurance policies. They must treat NHI and AID beneficiaries and receive fees as reimbursement for their costs. The different institutions receive different levels of reimbursement for those enrolled in the different schemes. For the same type of care, the fees, whether for ambulatory or inpatient care, are different between types of medical institutions; for example, the fees that tertiary care institutions receive are higher than those received by secondary care institutions, which are in turn higher than those received by primary care institutions [36]. Because fees per inpatient received by psychiatric hospitals are lower than those received by tertiary care institutions, most psychiatric hospitals with unoccupied beds have less incentive to discharge their psychiatric inpatients than tertiary care hospitals in order to compensate for the costs of maintaining beds. According to a further analysis of patient length of stay, those staying at psychiatric hospitals tended to stay 69 percent longer than patients staying at tertiary care hospitals, (details available on request). It can be hypothesized that a physician's practice style at psychiatric hospitals is affected by the psychosocial aspects of care [56].

### Ownership of institution

The private for-profit institutions have been favored due to their enhanced efficiency and competition, whereas non-profit institutions have been favored because of their lower costs and better quality [57,58]. In the present study, among AID beneficiaries, private institutions tended to be associated with larger MEs than did government institutions. One possible explanation for this is that private institutions provide more extensive psychiatric care than their government counterparts do. Another reason might be associated with the difference in the revenue structure between private and government institutions. Private institutions rely only on reimbursement revenue for their costs. In contrast, government institutions compensate for care expenses with reimbursement revenue as well as government subsidies. Therefore, private institutions, rather than government ones, would have a strong incentive to increase reimbursement revenue, thereby leading to higher patient MEs. Their efforts seem to be put more easily into practice for AID-enrolled psychiatric inpatients than for NHI-enrolled ones, because the coinsurance rate is much lower among AID beneficiaries than among NHI beneficiaries in Korea. Some Korean studies have demonstrated that psychiatric inpatients are likely to experience longer stays at private institutions than at public institutions [10,45].

### Patient composition and human resources

My results showed that several variables regarding the composition of inpatients with a psychiatric diagnosis were significantly associated with patient MEs. Among NHI beneficiaries, the proportions of inpatients with a psychiatric diagnosis that were male and of patients diagnosed into F0 or F2 categories were both positively associated with MEs. Similarly, among AID beneficiaries, the proportions of inpatients with a psychiatric diagnosis that were male and of psychiatric inpatients receiving AID exhibited a positive association with MEs. This suggests that patient MEs may be affected by some factors, which interact among patients, among physicians, or between patients and physicians within a medical institution [59-61]. Meanwhile, the variable representing human resources at an institution, Score 1, was negatively associated with patient MEs among AID beneficiaries. The finding that Score 1 increases with the number of professional staff members per institution standardized by the number of beds per institution suggests that if all other characteristics are equal, the more professionals that are employed by a medical institution, the lower psychiatric inpatient MEs. This result is different from a hypothesis of standard supplier-induced demand in the health care sector [62]. The influence of patient composition and human resources on psychiatric patient MEs needs to be further investigated.

### Study limitations

The present study is the first to quantitatively analyse the complete set of data for the entire population in a country, to investigate factors affecting psychiatric inpatient MEs and to differentiate those factors across different types of public health insurance programmes. Moreover, instead of standard linear regression analyses, multilevel analyses taking account of clustering within a medical institution were employed. Despite this obvious methodological advantage, several limitations should be mentioned. First, this claim-based study limits my ability to utilize such characteristics as pre-admission history and the severity of psychiatric illness. Second, because the data used in this study were collected during a particular period of time, my analyses do not consider psychiatric patient MEs for periods between the beginning of admission to an institution and inpatient discharge. However, psychiatric patient MEs during a particular period of time could be of importance, particularly from the perspective of mental health policy. Finally, the proportion of involuntary admissions or of readmission at a medical institution might affect patient ME, although this has not been identified in my datasets.

## Conclusions

According to the present study of psychiatric inpatient MEs, inpatient gender, sub-diagnosis, and institution type were more important among NHI beneficiaries than their AID counterparts. In contrast, both the age of the patient and the ownership of the institution were more important among AID beneficiaries than NHI beneficiaries. Therefore, the results of this study suggest that patient and institutional characteristics are associated with psychiatric inpatient MEs, and that these associations vary for beneficiaries of different public health insurance programmes. This suggests that policy efforts to reduce MEs of psychiatric inpatients staying at medical institutions should be made differently across the different types of public health insurance programmes.

## Abbreviations

(MEs): Medical Expenditures; (NHI): National Health Insurance; (AID): National Medical Care Aid; (ICD): International Classification of Diseases; (KRW): Korean Won.

## Competing interests

The author declares that they have no competing interests.

## Pre-publication history

The pre-publication history for this paper can be accessed here:

http://www.biomedcentral.com/1472-6963/10/263/prepub
